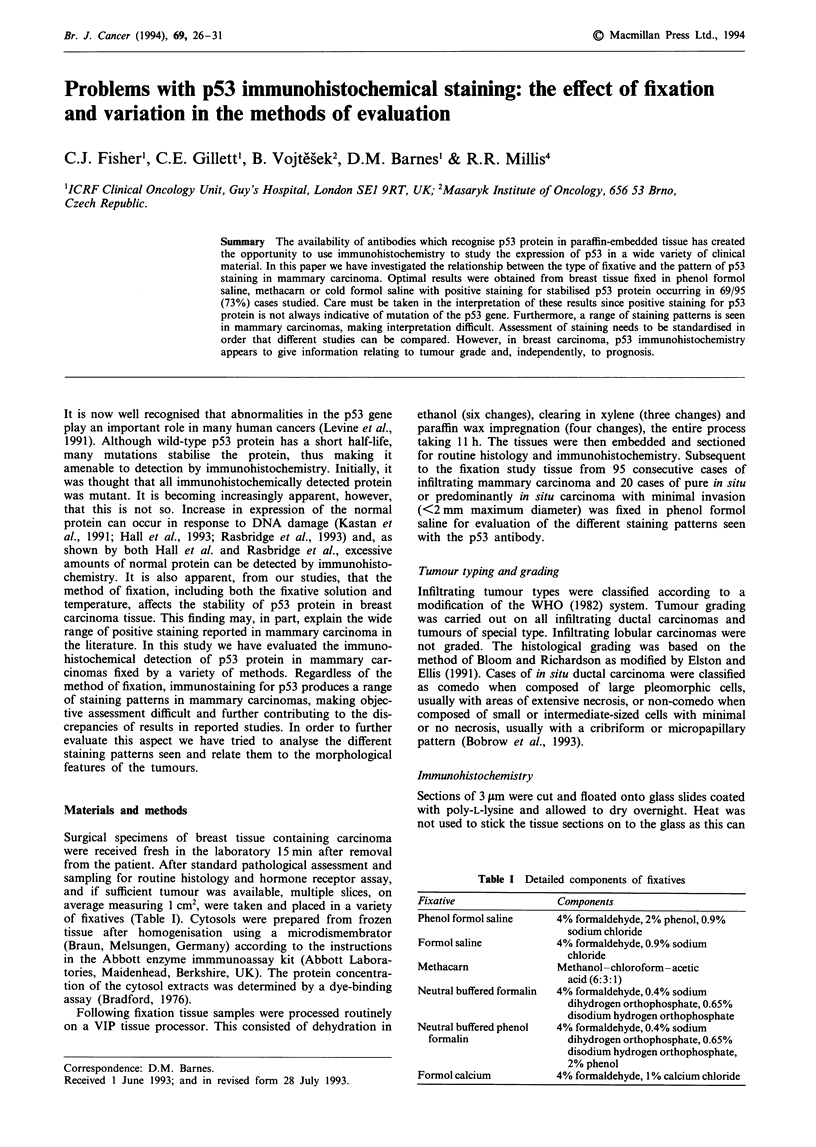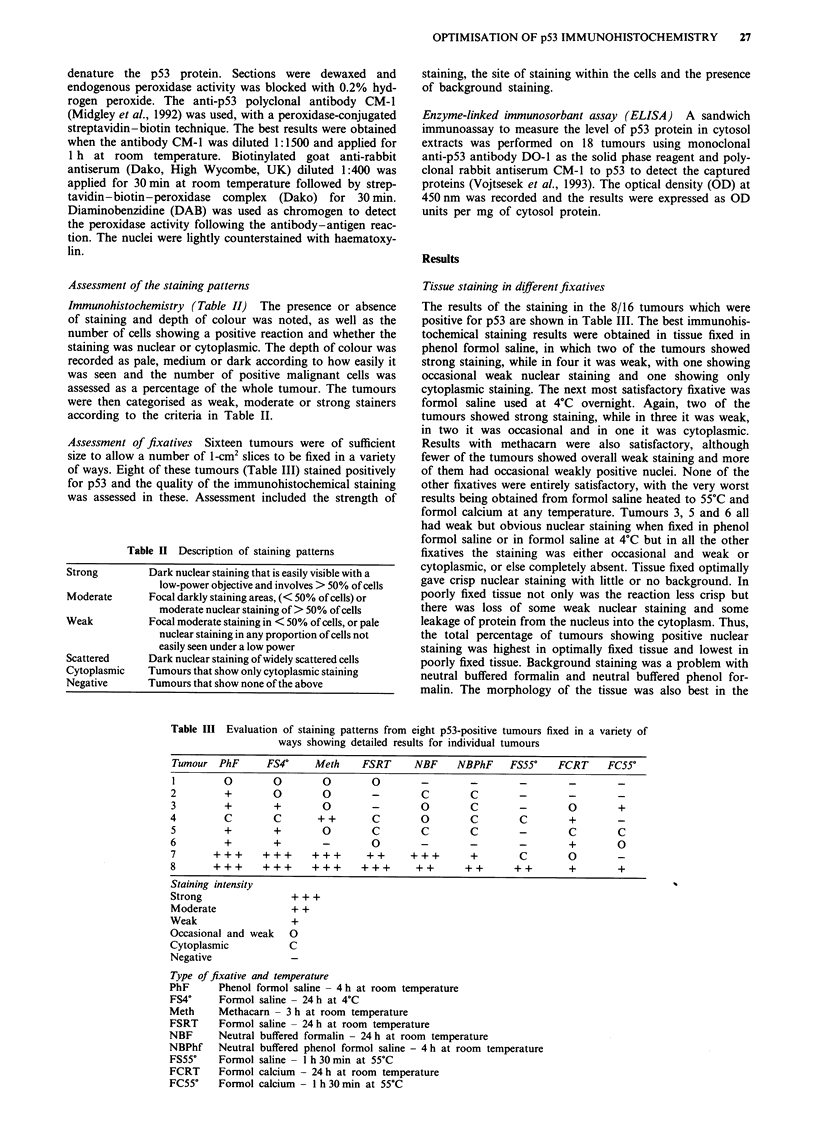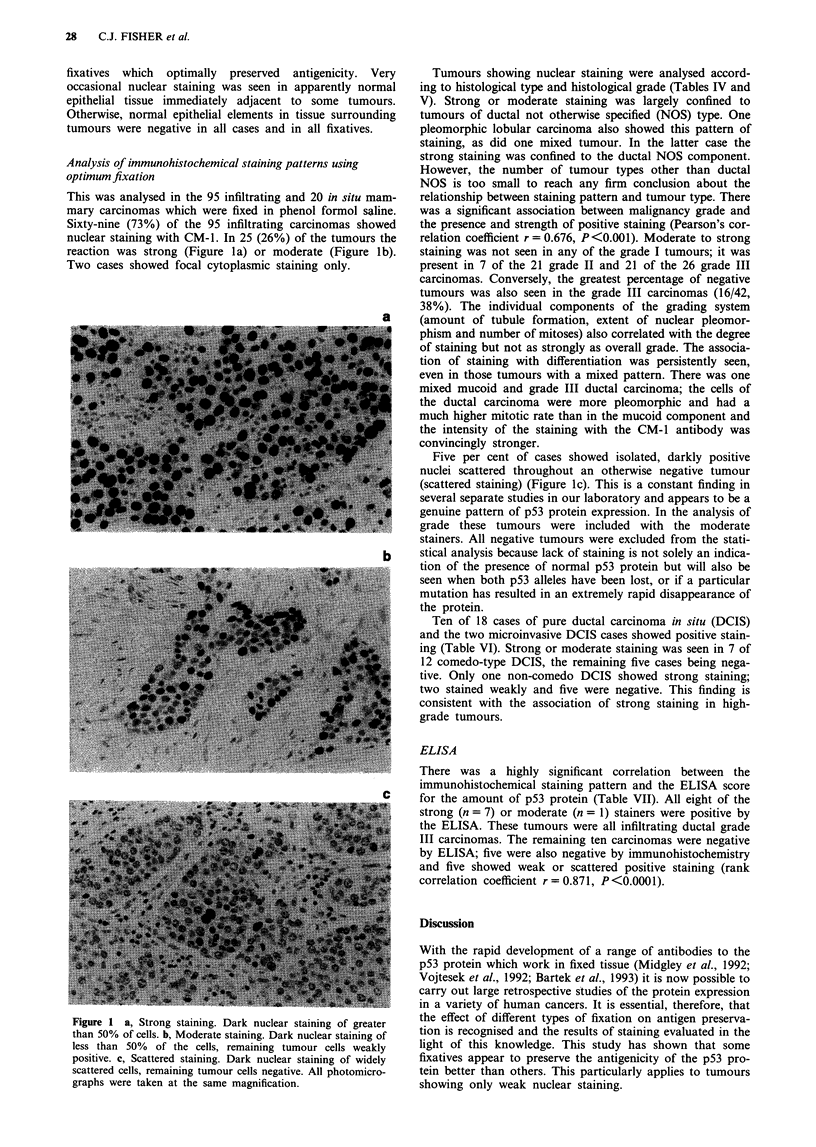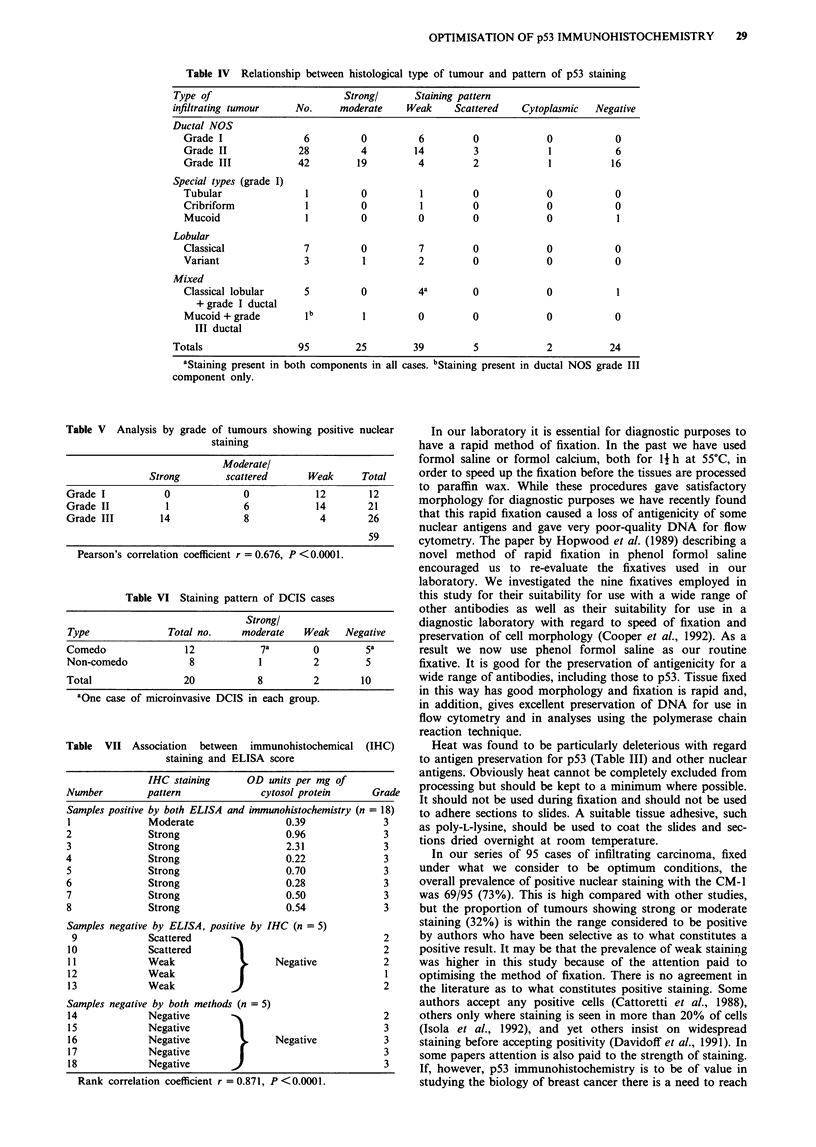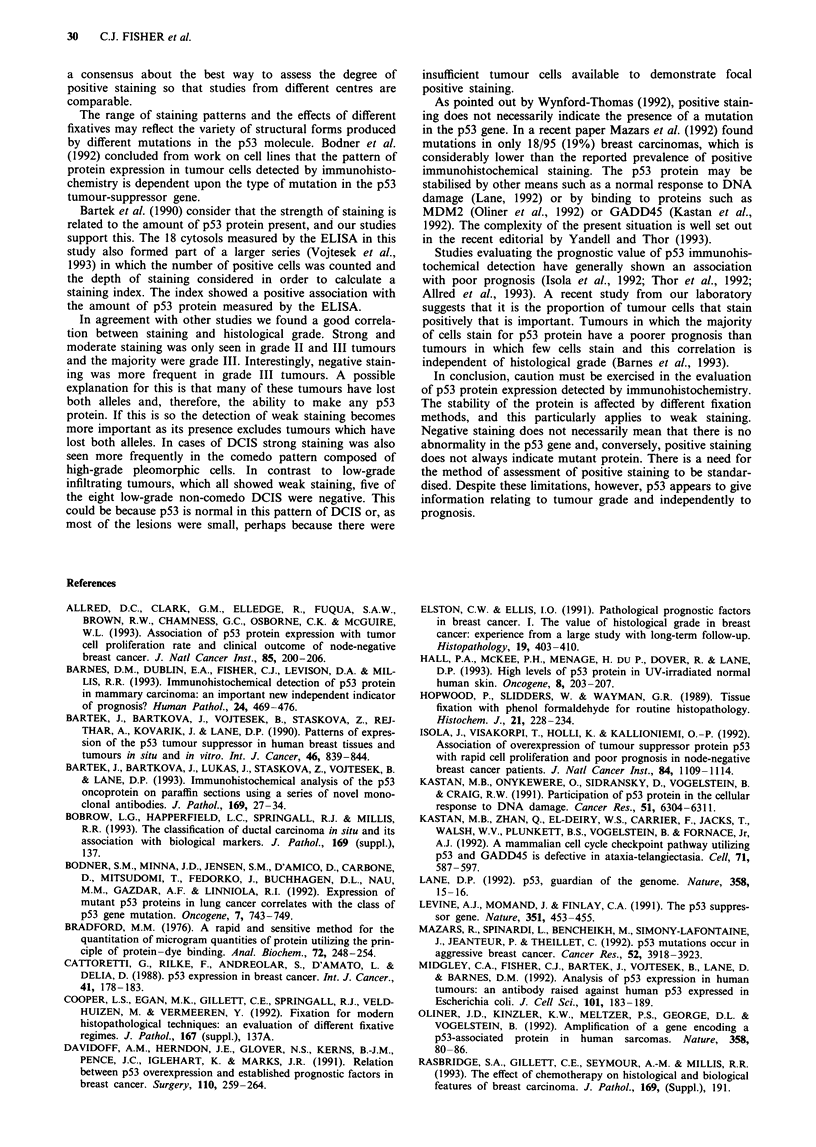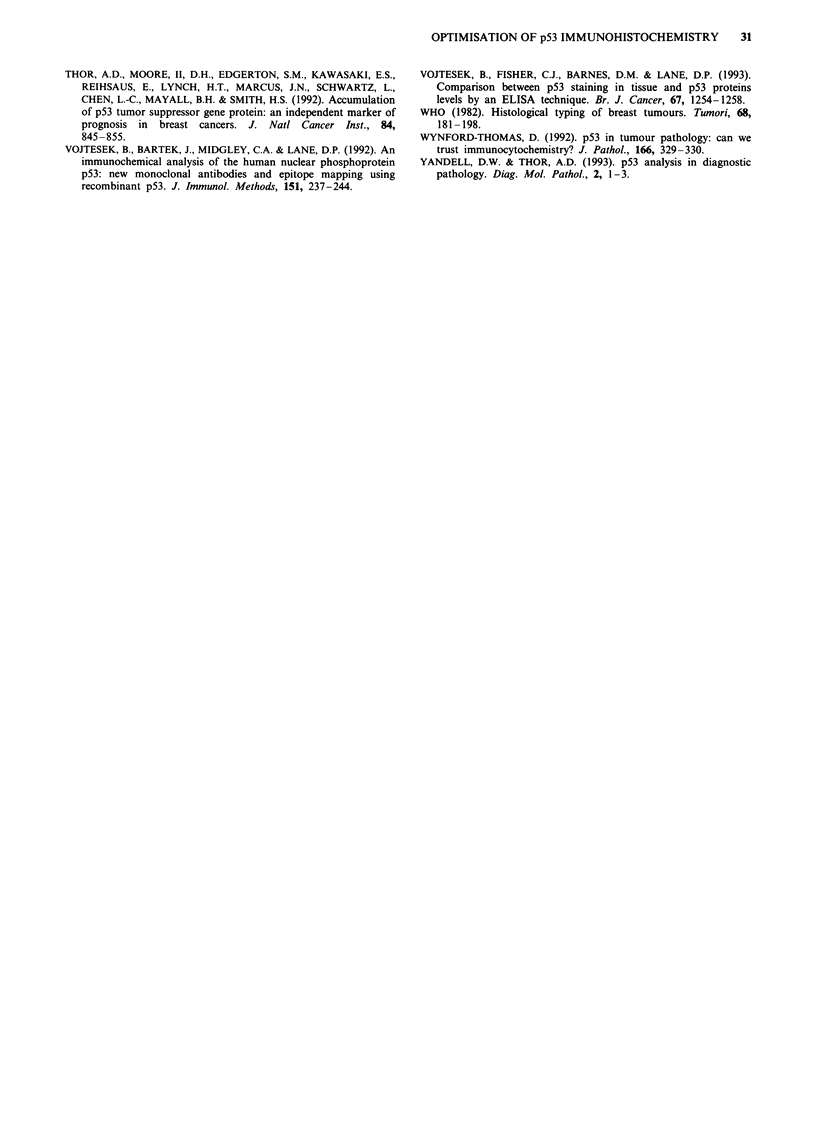# Problems with p53 immunohistochemical staining: the effect of fixation and variation in the methods of evaluation.

**DOI:** 10.1038/bjc.1994.4

**Published:** 1994-01

**Authors:** C. J. Fisher, C. E. Gillett, B. Vojtĕsek, D. M. Barnes, R. R. Millis

**Affiliations:** ICRF Clinical Oncology Unit, Guy's Hospital, London, UK.

## Abstract

**Images:**


Br. J. Cancer (1994), 69, 26-31 © Macmillan Press Ltd., 1994

### Problems with p53 immunohistochemical staining: the effect of fixation and variation in the methods of evaluation

C.J. Fisher[1], C.E. Gillett[1], B. Vojtěšek[2], D.M. Barnes[1] & R.R. Millis[4]

[1]*ICRF Clinical Oncology Unit, Guy's Hospital, London SE1 9RT, UK;* [2]*Masaryk Institute of Oncology, 656 53 Brno, Czech Republic.*

**Summary** The availability of antibodies which recognise p53 protein in paraffin-embedded tissue has created the opportunity to use immunohistochemistry to study the expression of p53 in a wide variety of clinical material. In this paper we have investigated the relationship between the type of fixative and the pattern of p53 staining in mammary carcinoma. Optimal results were obtained from breast tissue fixed in phenol formol saline, methacarn or cold formol saline with positive staining for stabilised p53 protein occurring in 69/95 (73%) cases studied. Care must be taken in the interpretation of these results since positive staining for p53 protein is not always indicative of mutation of the p53 gene. Furthermore, a range of staining patterns is seen in mammary carcinomas, making interpretation difficult. Assessment of staining needs to be standardised in order that different studies can be compared. However, in breast carcinoma, p53 immunohistochemistry appears to give information relating to tumour grade and, independently, to prognosis.

It is now well recognised that abnormalities in the p53 gene play an important role in many human cancers (Levine *et al.*, 1991). Although wild-type p53 protein has a short half-life, many mutations stabilise the protein, thus making it amenable to detection by immunohistochemistry. Initially, it was thought that all immunohistochemically detected protein was mutant. It is becoming increasingly apparent, however, that this is not so. Increase in expression of the normal protein can occur in response to DNA damage (Kastan *et al.*, 1991; Hall *et al.*, 1993; Rasbridge *et al.*, 1993) and, as shown by both Hall *et al.* and Rasbridge *et al.*, excessive amounts of normal protein can be detected by immunohistochemistry. It is also apparent, from our studies, that the method of fixation, including both the fixative solution and temperature, affects the stability of p53 protein in breast carcinoma tissue. This finding may, in part, explain the wide range of positive staining reported in mammary carcinoma in the literature. In this study we have evaluated the immunohistochemical detection of p53 protein in mammary carcinomas fixed by a variety of methods. Regardless of the method of fixation, immunostaining for p53 produces a range of staining patterns in mammary carcinomas, making objective assessment difficult and further contributing to the discrepancies of results in reported studies. In order to further evaluate this aspect we have tried to analyse the different staining patterns seen and relate them to the morphological features of the tumours.

### Materials and methods

Surgical specimens of breast tissue containing carcinoma were received fresh in the laboratory 15 min after removal from the patient. After standard pathological assessment and sampling for routine histology and hormone receptor assay, and if sufficient tumour was available, multiple slices, on average measuring 1 $cm^2$, were taken and placed in a variety of fixatives (Table I). Cytosols were prepared from frozen tissue after homogenisation using a microdismembrator (Braun, Melsungen, Germany) according to the instructions in the Abbott enzyme immmunoassay kit (Abbott Laboratories, Maidenhead, Berkshire, UK). The protein concentration of the cytosol extracts was determined by a dye-binding assay (Bradford, 1976).

Following fixation tissue samples were processed routinely on a VIP tissue processor. This consisted of dehydration in ethanol (six changes), clearing in xylene (three changes) and paraffin wax impregnation (four changes), the entire process taking 11 h. The tissues were then embedded and sectioned for routine histology and immunohistochemistry. Subsequent to the fixation study tissue from 95 consecutive cases of infiltrating mammary carcinoma and 20 cases of pure *in situ* or predominantly *in situ* carcinoma with minimal invasion (<2 mm maximum diameter) was fixed in phenol formol saline for evaluation of the different staining patterns seen with the p53 antibody.

### *Tumour typing and grading*

Infiltrating tumour types were classified according to a modification of the WHO (1982) system. Tumour grading was carried out on all infiltrating ductal carcinomas and tumours of special type. Infiltrating lobular carcinomas were not graded. The histological grading was based on the method of Bloom and Richardson as modified by Elston and Ellis (1991). Cases of *in situ* ductal carcinoma were classified as comedo when composed of large pleomorphic cells, usually with areas of extensive necrosis, or non-comedo when composed of small or intermediate-sized cells with minimal or no necrosis, usually with a cribriform or micropapillary pattern (Bobrow *et al.*, 1993).

### *Immunohistochemistry*

Sections of 3 μm were cut and floated onto glass slides coated with poly-L-lysine and allowed to dry overnight. Heat was not used to stick the tissue sections on to the glass as this can

**Table I** Detailed components of fixatives

| *Fixative* | *Components* |
|---|---|
| Phenol formol saline | 4% formaldehyde, 2% phenol, 0.9% sodium chloride |
| Formol saline | 4% formaldehyde, 0.9% sodium chloride |
| Methacarn | Methanol–chloroform–acetic acid (6:3:1) |
| Neutral buffered formalin | 4% formaldehyde, 0.4% sodium dihydrogen orthophosphate, 0.65% disodium hydrogen orthophosphate |
| Neutral buffered phenol formalin | 4% formaldehyde, 0.4% sodium dihydrogen orthophosphate, 0.65% disodium hydrogen orthophosphate, 2% phenol |
| Formol calcium | 4% formaldehyde, 1% calcium chloride |

Correspondence: D.M. Barnes.
Received 1 June 1993; and in revised form 28 July 1993.

denature the p53 protein. Sections were dewaxed and endogenous peroxidase activity was blocked with 0.2% hydrogen peroxide. The anti-p53 polyclonal antibody CM-1 (Midgley *et al.*, 1992) was used, with a peroxidase-conjugated streptavidin–biotin technique. The best results were obtained when the antibody CM-1 was diluted 1:1500 and applied for 1 h at room temperature. Biotinylated goat anti-rabbit antiserum (Dako, High Wycombe, UK) diluted 1:400 was applied for 30 min at room temperature followed by streptavidin–biotin–peroxidase complex (Dako) for 30 min. Diaminobenzidine (DAB) was used as chromogen to detect the peroxidase activity following the antibody–antigen reaction. The nuclei were lightly counterstained with haematoxylin.

### *Assessment of the staining patterns*

*Immunohistochemistry (Table II)* The presence or absence of staining and depth of colour was noted, as well as the number of cells showing a positive reaction and whether the staining was nuclear or cytoplasmic. The depth of colour was recorded as pale, medium or dark according to how easily it was seen and the number of positive malignant cells was assessed as a percentage of the whole tumour. The tumours were then categorised as weak, moderate or strong stainers according to the criteria in Table II.

*Assessment of fixatives* Sixteen tumours were of sufficient size to allow a number of 1-$cm^2$ slices to be fixed in a variety of ways. Eight of these tumours (Table III) stained positively for p53 and the quality of the immunohistochemical staining was assessed in these. Assessment included the strength of staining, the site of staining within the cells and the presence of background staining.

**Table II** Description of staining patterns

| | |
|---|---|
| Strong | Dark nuclear staining that is easily visible with a low-power objective and involves > 50% of cells |
| Moderate | Focal darkly staining areas, (< 50% of cells) or moderate nuclear staining of > 50% of cells |
| Weak | Focal moderate staining in < 50% of cells, or pale nuclear staining in any proportion of cells not easily seen under a low power |
| Scattered | Dark nuclear staining of widely scattered cells |
| Cytoplasmic | Tumours that show only cytoplasmic staining |
| Negative | Tumours that show none of the above |

*Enzyme-linked immunosorbant assay (ELISA)* A sandwich immunoassay to measure the level of p53 protein in cytosol extracts was performed on 18 tumours using monoclonal anti-p53 antibody DO-1 as the solid phase reagent and polyclonal rabbit antiserum CM-1 to p53 to detect the captured proteins (Vojtsesek *et al.*, 1993). The optical density (OD) at 450 nm was recorded and the results were expressed as OD units per mg of cytosol protein.

### Results

### *Tissue staining in different fixatives*

The results of the staining in the 8/16 tumours which were positive for p53 are shown in Table III. The best immunohistochemical staining results were obtained in tissue fixed in phenol formol saline, in which two of the tumours showed strong staining, while in four it was weak, with one showing occasional weak nuclear staining and one showing only cytoplasmic staining. The next most satisfactory fixative was formol saline used at 4°C overnight. Again, two of the tumours showed strong staining, while in three it was weak, in two it was occasional and in one it was cytoplasmic. Results with methacarn were also satisfactory, although fewer of the tumours showed overall weak staining and more of them had occasional weakly positive nuclei. None of the other fixatives were entirely satisfactory, with the very worst results being obtained from formol saline heated to 55°C and formol calcium at any temperature. Tumours 3, 5 and 6 all had weak but obvious nuclear staining when fixed in phenol formol saline or in formol saline at 4°C but in all the other fixatives the staining was either occasional and weak or cytoplasmic, or else completely absent. Tissue fixed optimally gave crisp nuclear staining with little or no background. In poorly fixed tissue not only was the reaction less crisp but there was loss of some weak nuclear staining and some leakage of protein from the nucleus into the cytoplasm. Thus, the total percentage of tumours showing positive nuclear staining was highest in optimally fixed tissue and lowest in poorly fixed tissue. Background staining was a problem with neutral buffered formalin and neutral buffered phenol formalin. The morphology of the tissue was also best in the

**Table III** Evaluation of staining patterns from eight p53-positive tumours fixed in a variety of ways showing detailed results for individual tumours

| *Tumour* | *PhF* | *FS4°* | *Meth* | *FSRT* | *NBF* | *NBPhF* | *FS55°* | *FCRT* | *FC55°* |
|---|---|---|---|---|---|---|---|---|---|
| 1 | O | O | O | O | – | – | – | – | – |
| 2 | + | O | O | – | C | C | – | – | – |
| 3 | + | + | O | – | O | C | – | O | + |
| 4 | C | C | ++ | C | O | C | C | + | – |
| 5 | + | + | O | C | C | C | – | C | C |
| 6 | + | + | – | O | – | – | – | + | O |
| 7 | +++ | +++ | +++ | ++ | +++ | + | C | O | – |
| 8 | +++ | +++ | +++ | +++ | ++ | ++ | ++ | + | + |

*Staining intensity*

| | |
|---|---|
| Strong | +++ |
| Moderate | ++ |
| Weak | + |
| Occasional and weak | O |
| Cytoplasmic | C |
| Negative | – |

*Type of fixative and temperature*

| | |
|---|---|
| PhF | Phenol formol saline – 4 h at room temperature |
| FS4° | Formol saline – 24 h at 4°C |
| Meth | Methacarn – 3 h at room temperature |
| FSRT | Formol saline – 24 h at room temperature |
| NBF | Neutral buffered formalin – 24 h at room temperature |
| NBPhf | Neutral buffered phenol formol saline – 4 h at room temperature |
| FS55° | Formol saline – 1 h 30 min at 55°C |
| FCRT | Formol calcium – 24 h at room temperature |
| FC55° | Formol calcium – 1 h 30 min at 55°C |

fixatives which optimally preserved antigenicity. Very occasional nuclear staining was seen in apparently normal epithelial tissue immediately adjacent to some tumours. Otherwise, normal epithelial elements in tissue surrounding tumours were negative in all cases and in all fixatives.

*Analysis of immunohistochemical staining patterns using optimum fixation*

This was analysed in the 95 infiltrating and 20 *in situ* mammary carcinomas which were fixed in phenol formol saline. Sixty-nine (73%) of the 95 infiltrating carcinomas showed nuclear staining with CM-1. In 25 (26%) of the tumours the reaction was strong (Figure 1a) or moderate (Figure 1b). Two cases showed focal cytoplasmic staining only.

**Figure 1** **a**, Strong staining. Dark nuclear staining of greater than 50% of cells. **b**, Moderate staining. Dark nuclear staining of less than 50% of the cells, remaining tumour cells weakly positive. **c**, Scattered staining. Dark nuclear staining of widely scattered cells, remaining tumour cells negative. All photomicrographs were taken at the same magnification.

Tumours showing nuclear staining were analysed according to histological type and histological grade (Tables IV and V). Strong or moderate staining was largely confined to tumours of ductal not otherwise specified (NOS) type. One pleomorphic lobular carcinoma also showed this pattern of staining, as did one mixed tumour. In the latter case the strong staining was confined to the ductal NOS component. However, the number of tumour types other than ductal NOS is too small to reach any firm conclusion about the relationship between staining pattern and tumour type. There was a significant association between malignancy grade and the presence and strength of positive staining (Pearson's correlation coefficient $r = 0.676$, $P < 0.001$). Moderate to strong staining was not seen in any of the grade I tumours; it was present in 7 of the 21 grade II and 21 of the 26 grade III carcinomas. Conversely, the greatest percentage of negative tumours was also seen in the grade III carcinomas (16/42, 38%). The individual components of the grading system (amount of tubule formation, extent of nuclear pleomorphism and number of mitoses) also correlated with the degree of staining but not as strongly as overall grade. The association of staining with differentiation was persistently seen, even in those tumours with a mixed pattern. There was one mixed mucoid and grade III ductal carcinoma; the cells of the ductal carcinoma were more pleomorphic and had a much higher mitotic rate than in the mucoid component and the intensity of the staining with the CM-1 antibody was convincingly stronger.

Five per cent of cases showed isolated, darkly positive nuclei scattered throughout an otherwise negative tumour (scattered staining) (Figure 1c). This is a constant finding in several separate studies in our laboratory and appears to be a genuine pattern of p53 protein expression. In the analysis of grade these tumours were included with the moderate stainers. All negative tumours were excluded from the statistical analysis because lack of staining is not solely an indication of the presence of normal p53 protein but will also be seen when both p53 alleles have been lost, or if a particular mutation has resulted in an extremely rapid disappearance of the protein.

Ten of 18 cases of pure ductal carcinoma *in situ* (DCIS) and the two microinvasive DCIS cases showed positive staining (Table VI). Strong or moderate staining was seen in 7 of 12 comedo-type DCIS, the remaining five cases being negative. Only one non-comedo DCIS showed strong staining; two stained weakly and five were negative. This finding is consistent with the association of strong staining in high-grade tumours.

*ELISA*

There was a highly significant correlation between the immunohistochemical staining pattern and the ELISA score for the amount of p53 protein (Table VII). All eight of the strong ($n = 7$) or moderate ($n = 1$) stainers were positive by the ELISA. These tumours were all infiltrating ductal grade III carcinomas. The remaining ten carcinomas were negative by ELISA; five were also negative by immunohistochemistry and five showed weak or scattered positive staining (rank correlation coefficient $r = 0.871$, $P < 0.0001$).

### Discussion

With the rapid development of a range of antibodies to the p53 protein which work in fixed tissue (Midgley *et al.*, 1992; Vojtesek *et al.*, 1992; Bartek *et al.*, 1993) it is now possible to carry out large retrospective studies of the protein expression in a variety of human cancers. It is essential, therefore, that the effect of different types of fixation on antigen preservation is recognised and the results of staining evaluated in the light of this knowledge. This study has shown that some fixatives appear to preserve the antigenicity of the p53 protein better than others. This particularly applies to tumours showing only weak nuclear staining.

**Table IV** Relationship between histological type of tumour and pattern of p53 staining

| *Type of infiltrating tumour* | *No.* | *Staining pattern* *Strong/ moderate* | *Weak* | *Scattered* | *Cytoplasmic* | *Negative* |
|---|---|---|---|---|---|---|
| *Ductal NOS* | | | | | | |
| Grade I | 6 | 0 | 6 | 0 | 0 | 0 |
| Grade II | 28 | 4 | 14 | 3 | 1 | 6 |
| Grade III | 42 | 19 | 4 | 2 | 1 | 16 |
| *Special types* (grade I) | | | | | | |
| Tubular | 1 | 0 | 1 | 0 | 0 | 0 |
| Cribriform | 1 | 0 | 1 | 0 | 0 | 0 |
| Mucoid | 1 | 0 | 0 | 0 | 0 | 1 |
| *Lobular* | | | | | | |
| Classical | 7 | 0 | 7 | 0 | 0 | 0 |
| Variant | 3 | 1 | 2 | 0 | 0 | 0 |
| *Mixed* | | | | | | |
| Classical lobular + grade I ductal | 5 | 0 | 4[a] | 0 | 0 | 1 |
| Mucoid + grade III ductal | 1[b] | 1 | 0 | 0 | 0 | 0 |
| Totals | 95 | 25 | 39 | 5 | 2 | 24 |

[a]Staining present in both components in all cases. [b]Staining present in ductal NOS grade III component only.

**Table V** Analysis by grade of tumours showing positive nuclear staining

| | *Strong* | *Moderate/ scattered* | *Weak* | *Total* |
|---|---|---|---|---|
| Grade I | 0 | 0 | 12 | 12 |
| Grade II | 1 | 6 | 14 | 21 |
| Grade III | 14 | 8 | 4 | 26 |
| | | | | 59 |

Pearson's correlation coefficient $r = 0.676$, $P < 0.0001$.

**Table VI** Staining pattern of DCIS cases

| *Type* | *Total no.* | *Strong/ moderate* | *Weak* | *Negative* |
|---|---|---|---|---|
| Comedo | 12 | 7[a] | 0 | 5[a] |
| Non-comedo | 8 | 1 | 2 | 5 |
| Total | 20 | 8 | 2 | 10 |

[a]One case of microinvasive DCIS in each group.

**Table VII** Association between immunohistochemical (IHC) staining and ELISA score

| *Number* | *IHC staining pattern* | *OD units per mg of cytosol protein* | *Grade* |
|---|---|---|---|
| *Samples positive by both ELISA and immunohistochemistry* (*n* = 18) | | | |
| 1 | Moderate | 0.39 | 3 |
| 2 | Strong | 0.96 | 3 |
| 3 | Strong | 2.31 | 3 |
| 4 | Strong | 0.22 | 3 |
| 5 | Strong | 0.70 | 3 |
| 6 | Strong | 0.28 | 3 |
| 7 | Strong | 0.50 | 3 |
| 8 | Strong | 0.54 | 3 |
| *Samples negative by ELISA, positive by IHC* (*n* = 5) | | | |
| 9 | Scattered | | 2 |
| 10 | Scattered | | 2 |
| 11 | Weak | Negative | 2 |
| 12 | Weak | | 1 |
| 13 | Weak | | 2 |
| *Samples negative by both methods* (*n* = 5) | | | |
| 14 | Negative | | 2 |
| 15 | Negative | | 3 |
| 16 | Negative | Negative | 3 |
| 17 | Negative | | 3 |
| 18 | Negative | | 3 |

Rank correlation coefficient $r = 0.871$, $P < 0.0001$.

In our laboratory it is essential for diagnostic purposes to have a rapid method of fixation. In the past we have used formol saline or formol calcium, both for 1½ h at 55°C, in order to speed up the fixation before the tissues are processed to paraffin wax. While these procedures gave satisfactory morphology for diagnostic purposes we have recently found that this rapid fixation caused a loss of antigenicity of some nuclear antigens and gave very poor-quality DNA for flow cytometry. The paper by Hopwood *et al.* (1989) describing a novel method of rapid fixation in phenol formol saline encouraged us to re-evaluate the fixatives used in our laboratory. We investigated the nine fixatives employed in this study for their suitability for use with a wide range of other antibodies as well as their suitability for use in a diagnostic laboratory with regard to speed of fixation and preservation of cell morphology (Cooper *et al.*, 1992). As a result we now use phenol formol saline as our routine fixative. It is good for the preservation of antigenicity for a wide range of antibodies, including those to p53. Tissue fixed in this way has good morphology and fixation is rapid and, in addition, gives excellent preservation of DNA for use in flow cytometry and in analyses using the polymerase chain reaction technique.

Heat was found to be particularly deleterious with regard to antigen preservation for p53 (Table III) and other nuclear antigens. Obviously heat cannot be completely excluded from processing but should be kept to a minimum where possible. It should not be used during fixation and should not be used to adhere sections to slides. A suitable tissue adhesive, such as poly-L-lysine, should be used to coat the slides and sections dried overnight at room temperature.

In our series of 95 cases of infiltrating carcinoma, fixed under what we consider to be optimum conditions, the overall prevalence of positive nuclear staining with the CM-1 was 69/95 (73%). This is high compared with other studies, but the proportion of tumours showing strong or moderate staining (32%) is within the range considered to be positive by authors who have been selective as to what constitutes a positive result. It may be that the prevalence of weak staining was higher in this study because of the attention paid to optimising the method of fixation. There is no agreement in the literature as to what constitutes positive staining. Some authors accept any positive cells (Cattoretti *et al.*, 1988), others only where staining is seen in more than 20% of cells (Isola *et al.*, 1992), and yet others insist on widespread staining before accepting positivity (Davidoff *et al.*, 1991). In some papers attention is also paid to the strength of staining. If, however, p53 immunohistochemistry is to be of value in studying the biology of breast cancer there is a need to reach a consensus about the best way to assess the degree of positive staining so that studies from different centres are comparable.

The range of staining patterns and the effects of different fixatives may reflect the variety of structural forms produced by different mutations in the p53 molecule. Bodner *et al.* (1992) concluded from work on cell lines that the pattern of protein expression in tumour cells detected by immunohistochemistry is dependent upon the type of mutation in the p53 tumour-suppressor gene.

Bartek *et al.* (1990) consider that the strength of staining is related to the amount of p53 protein present, and our studies support this. The 18 cytosols measured by the ELISA in this study also formed part of a larger series (Vojtesek *et al.*, 1993) in which the number of positive cells was counted and the depth of staining considered in order to calculate a staining index. The index showed a positive association with the amount of p53 protein measured by the ELISA.

In agreement with other studies we found a good correlation between staining and histological grade. Strong and moderate staining was only seen in grade II and III tumours and the majority were grade III. Interestingly, negative staining was more frequent in grade III tumours. A possible explanation for this is that many of these tumours have lost both alleles and, therefore, the ability to make any p53 protein. If this is so the detection of weak staining becomes more important as its presence excludes tumours which have lost both alleles. In cases of DCIS strong staining was also seen more frequently in the comedo pattern composed of high-grade pleomorphic cells. In contrast to low-grade infiltrating tumours, which all showed weak staining, five of the eight low-grade non-comedo DCIS were negative. This could be because p53 is normal in this pattern of DCIS or, as most of the lesions were small, perhaps because there were insufficient tumour cells available to demonstrate focal positive staining.

As pointed out by Wynford-Thomas (1992), positive staining does not necessarily indicate the presence of a mutation in the p53 gene. In a recent paper Mazars *et al.* (1992) found mutations in only 18/95 (19%) breast carcinomas, which is considerably lower than the reported prevalence of positive immunohistochemical staining. The p53 protein may be stabilised by other means such as a normal response to DNA damage (Lane, 1992) or by binding to proteins such as MDM2 (Oliner *et al.*, 1992) or GADD45 (Kastan *et al.*, 1992). The complexity of the present situation is well set out in the recent editorial by Yandell and Thor (1993).

Studies evaluating the prognostic value of p53 immunohistochemical detection have generally shown an association with poor prognosis (Isola *et al.*, 1992; Thor *et al.*, 1992; Allred *et al.*, 1993). A recent study from our laboratory suggests that it is the proportion of tumour cells that stain positively that is important. Tumours in which the majority of cells stain for p53 protein have a poorer prognosis than tumours in which few cells stain and this correlation is independent of histological grade (Barnes *et al.*, 1993).

In conclusion, caution must be exercised in the evaluation of p53 protein expression detected by immunohistochemistry. The stability of the protein is affected by different fixation methods, and this particularly applies to weak staining. Negative staining does not necessarily mean that there is no abnormality in the p53 gene and, conversely, positive staining does not always indicate mutant protein. There is a need for the method of assessment of positive staining to be standardised. Despite these limitations, however, p53 appears to give information relating to tumour grade and independently to prognosis.

### References

ALLRED, D.C., CLARK, G.M., ELLEDGE, R., FUQUA, S.A.W., BROWN, R.W., CHAMNESS, G.C., OSBORNE, C.K. & MCGUIRE, W.L. (1993). Association of p53 protein expression with tumor cell proliferation rate and clinical outcome of node-negative breast cancer. *J. Natl Cancer Inst.*, **85**, 200-206.

BARNES, D.M., DUBLIN, E.A., FISHER, C.J., LEVISON, D.A. & MILLIS, R.R. (1993). Immunohistochemical detection of p53 protein in mammary carcinoma: an important new independent indicator of prognosis? *Human Pathol.*, **24**, 469-476.

BARTEK, J., BARTKOVA, J., VOJTESEK, B., STASKOVA, Z., REJTHAR, A., KOVARIK, J. & LANE, D.P. (1990). Patterns of expression of the p53 tumour suppressor in human breast tissues and tumours *in situ* and *in vitro*. *Int. J. Cancer*, **46**, 839-844.

BARTEK, J., BARTKOVA, J., LUKAS, J., STASKOVA, Z., VOJTESEK, B. & LANE, D.P. (1993). Immunohistochemical analysis of the p53 oncoprotein on paraffin sections using a series of novel monoclonal antibodies. *J. Pathol.*, **169**, 27-34.

BOBROW, L.G., HAPPERFIELD, L.C., SPRINGALL, R.J. & MILLIS, R.R. (1993). The classification of ductal carcinoma *in situ* and its association with biological markers. *J. Pathol.*, **169** (suppl.), 137.

BODNER, S.M., MINNA, J.D., JENSEN, S.M., D'AMICO, D., CARBONE, D., MITSUDOMI, T., FEDORKO, J., BUCHHAGEN, D.L., NAU, M.M., GAZDAR, A.F. & LINNIOLA, R.I. (1992). Expression of mutant p53 proteins in lung cancer correlates with the class of p53 gene mutation. *Oncogene*, **7**, 743-749.

BRADFORD, M.M. (1976). A rapid and sensitive method for the quantitation of microgram quantities of protein utilizing the principle of protein-dye binding. *Anal. Biochem.*, **72**, 248-254.

CATTORETTI, G., RILKE, F., ANDREOLAR, S., D'AMATO, L. & DELIA, D. (1988). p53 expression in breast cancer. *Int. J. Cancer.*, **41**, 178-183.

COOPER, L.S., EGAN, M.K., GILLETT, C.E., SPRINGALL, R.J., VELDHUIZEN, M. & VERMEEREN, Y. (1992). Fixation for modern histopathological techniques: an evaluation of different fixative regimes. *J. Pathol.*, **167** (suppl.), 137A.

DAVIDOFF, A.M., HERNDON, J.E., GLOVER, N.S., KERNS, B.-J.M., PENCE, J.C., IGLEHART, K. & MARKS, J.R. (1991). Relation between p53 overexpression and established prognostic factors in breast cancer. *Surgery*, **110**, 259-264.

ELSTON, C.W. & ELLIS, I.O. (1991). Pathological prognostic factors in breast cancer. I. The value of histological grade in breast cancer: experience from a large study with long-term follow-up. *Histopathology*, **19**, 403-410.

HALL, P.A., MCKEE, P.H., MENAGE, H. DU P., DOVER, R. & LANE, D.P. (1993). High levels of p53 protein in UV-irradiated normal human skin. *Oncogene*, **8**, 203-207.

HOPWOOD, P., SLIDDERS, W. & WAYMAN, G.R. (1989). Tissue fixation with phenol formaldehyde for routine histopathology. *Histochem. J.*, **21**, 228-234.

ISOLA, J., VISAKORPI, T., HOLLI, K. & KALLIONIEMI, O.-P. (1992). Association of overexpression of tumour suppressor protein p53 with rapid cell proliferation and poor prognosis in node-negative breast cancer patients. *J. Natl Cancer Inst.*, **84**, 1109-1114.

KASTAN, M.B., ONYKEWERE, O., SIDRANSKY, D., VOGELSTEIN, B. & CRAIG, R.W. (1991). Participation of p53 protein in the cellular response to DNA damage. *Cancer Res.*, **51**, 6304-6311.

KASTAN, M.B., ZHAN, Q., EL-DEIRY, W.S., CARRIER, F., JACKS, T., WALSH, W.V., PLUNKETT, B.S., VOGELSTEIN, B. & FORNACE, Jr, A.J. (1992). A mammalian cell cycle checkpoint pathway utilizing p53 and GADD45 is defective in ataxia-telangiectasia. *Cell*, **71**, 587-597.

LANE, D.P. (1992). p53, guardian of the genome. *Nature*, **358**, 15-16.

LEVINE, A.J., MOMAND, J. & FINLAY, C.A. (1991). The p53 suppressor gene. *Nature*, **351**, 453-455.

MAZARS, R., SPINARDI, L., BENCHEIKH, M., SIMONY-LAFONTAINE, J., JEANTEUR, P. & THEILLET, C. (1992). p53 mutations occur in aggressive breast cancer. *Cancer Res.*, **52**, 3918-3923.

MIDGLEY, C.A., FISHER, C.J., BARTEK, J., VOJTESEK, B., LANE, D. & BARNES, D.M. (1992). Analysis of p53 expression in human tumours: an antibody raised against human p53 expressed in Escherichia coli. *J. Cell Sci.*, **101**, 183-189.

OLINER, J.D., KINZLER, K.W., MELTZER, P.S., GEORGE, D.L. & VOGELSTEIN, B. (1992). Amplification of a gene encoding a p53-associated protein in human sarcomas. *Nature*, **358**, 80-86.

RASBRIDGE, S.A., GILLETT, C.E., SEYMOUR, A.-M. & MILLIS, R.R. (1993). The effect of chemotherapy on histological and biological features of breast carcinoma. *J. Pathol.*, **169**, (Suppl.), 191.

THOR, A.D., MOORE, II, D.H., EDGERTON, S.M., KAWASAKI, E.S., REIHSAUS, E., LYNCH, H.T., MARCUS, J.N., SCHWARTZ, L., CHEN, L.-C., MAYALL, B.H. & SMITH, H.S. (1992). Accumulation of p53 tumor suppressor gene protein: an independent marker of prognosis in breast cancers. *J. Natl Cancer Inst.*, **84**, 845–855.

VOJTESEK, B., BARTEK, J., MIDGLEY, C.A. & LANE, D.P. (1992). An immunochemical analysis of the human nuclear phosphoprotein p53: new monoclonal antibodies and epitope mapping using recombinant p53. *J. Immunol. Methods*, **151**, 237–244.

VOJTESEK, B., FISHER, C.J., BARNES, D.M. & LANE, D.P. (1993). Comparison between p53 staining in tissue and p53 proteins levels by an ELISA technique. *Br. J. Cancer*, **67**, 1254–1258.

WHO (1982). Histological typing of breast tumours. *Tumori*, **68**, 181–198.

WYNFORD-THOMAS, D. (1992). p53 in tumour pathology: can we trust immunocytochemistry? *J. Pathol.*, **166**, 329–330.

YANDELL, D.W. & THOR, A.D. (1993). p53 analysis in diagnostic pathology. *Diag. Mol. Pathol.*, **2**, 1–3.